# Rituximab is Indispensable for Pediatric Heart Transplant Recipients Developing Post Transplant Lymphoproliferative Disorders

**Published:** 2013-07-22

**Authors:** R Karbasi-Afshar, S Taheri

**Affiliations:** 1 Cardiovascular Research Center; Baqiyatallah University of Medical Sciences, Tehran, Iran.; 2 Medical Research Group, Tehran, Iran.

**Keywords:** Pediatrics, Heart Transplantation, rituximab

## Abstract

Rituximab, an anti-CD20 agent, has been suggested as an effective strategy to deal with post transplant lymphoproliferative disorders (PTLD). In the current study, we aim to evaluate the efficacy of rituximab therapy in heart transplant population developing PTLD. A comprehensive search of the literature was performed to gather the available data on lymphoproliferative disorders occurring in heart transplant patients. Finally, data of 125 patients from 26 previously published studies were included into the study. Patients who underwent rituximab therapy had significantly worse tumoral histopathology features (P-value= 0.003).

Survival analyses showed no significant difference regarding receiving rituximab therapy for heart recipients; however, when the analysis was repeated only including data of pediatric patients, significant beneficial effects for pediatric were found for rituximab therapy. In fact, no children undergoing rituximab therapy died during the follow up. In conclusion, this study showed that rituximab therapy in pediatric heart transplant recipients with PTLD represents surprisingly excellent results, making rituximab an indispensable agent in the management of the disease. To define feasibility of rituximab therapy in adult recipients of heart graft with PTLD, randomized controlled trials are needed.

## Introduction

The science of organ transplantation has witnessed substantial succession through the recent two to three decades, after the significant advances achieved either in the surgical techniques or introduction of highly potent immunosuppressive agents for preventing rejection episodes. These advances have provided the transplantation practice, an opportunity to experience success in transplanting different organs of the body, even critical organs like the heart, for which rather unfavorable results used to be obtained before the recent progressions. On the other hand, the widespread use of powerful immunosuppression for preventing the risk of rejection has emerged new troubles that can adversely affect patients’ survival in different ways (1-3). Post transplant lymphoproliferative disorders (PTLD are one of the most frequent and also fatal disorders that have been reported as a consequence of immunosuppressive therapy. The vulnerability to the development of lymphomas in organ transplant recipients has firstly been reported by Penn et al. (4) in 1969, characterized by lymphoid proliferation of B- or T-cell origins in different organs. Then, after a large amount of reports from all over the world, the high incidence of PTLD among recipients of all types of organs indicated, either in adults or children, and poor outcome (5-9). Different studies have suggested that the incidence, time interval, prognosis, and presentation of PTLD vary depending on several factors including the demographics of the patients, the immunosuppression employed, and the type of organ transplanted (10-12). The reported incidence of PTLD in heart transplant recipients varies from 2% to up to 25% with higher rates in pediatric settings and EBV negative patients (13, 14). 

Since the majority of PTLD lesions arise from B lymphocytes, anti-B monoclonal antibodies seem logical agents for administration; Rituximab, an anti-CD20 monoclonal antibody, has been used to treat non-Hodgkin’s lymphoma, and had resulted in a favorable outcome (15). In the transplant context, this agent prevents the risk of graft rejection due to immunosuppression discontinuation, and minimizes the systemic toxicity secondary to chemotherapy. Therefore, rituximab administration concomitant to reducing immunosuppression has been suggested as an effective strategy to deal with PTLD (16, 17). In the current study, we aim to evaluate the efficacy of rituximab therapy in a heart transplant population developing PTLD.

## Methods and material

**Approach to the study**

We conducted a comprehensive search by Pubmed and Google Scholar for the available data in lymphoproliferative disorders occurring in heart transplant patients. Keywords used included “lymphoproliferative disorders + transplantation + heart + survival” “lymphoproliferative disorders + transplantation + heart + outcome” “lymphoproliferative disorders + heart transplantation + mortality” “lymphoproliferative disorder + transplantation + heart + treatment” “PTLD + heart + survival” “PTLD + heart + outcome”, and similar combination of the keywords. In cases that we were not able to achieve the full text of the articles, emails were sent to corresponding authors requesting the article. To minimize selection bias, we only included studies reporting their series of patients from single or multi center populations, and studies with any specific selection criterion were excluded from the analysis. Finally, data from 26 previously published studies from various countries (18-43) were included into the study. Patients whose PTLD presentation time was within the first 12 months post transplantation were considered as “early-onset PTLD” group, and the heart graft recipients who represented the disease beyond this time period after transplantation were categorized as “late onset PTLD” patients.

Study population

Overall, 125 recipients of cardiac allograft were included into the study. 34 (27.2%) of the study population were patients who had been received rituximab after PTLD diagnosis while the remaining 91 (72.8%) patients had been confirmed not having gotten rituximab. 

**Data standardization and terminology definition**

Because of inconsistencies in the data presentation by different included reports, we had to standardize data of different measures to cumulate into a unique database. Disseminated lymphoma was diagnosed when it was declared by the authors or at least three different organs (excluding different lymph node areas) were involved by PTLD, reported in 21 (27.6%; 49 unreported) of patients. Multi organ involvement defined as involvement of more than a unique organ as well as more than one lymphatic region was diagnosed in 34 (40%; 40 unreported) patients. 

**Response to treatment**

Response to treatment was defined as any favorable change in the cancer measures as well as patients’ clinical condition termed as “remission”, and was defined when declared by authors or when patients were alive after their 24th month of PTLD diagnosis, and no remission was defined when a patient dies within the first month post PTLD diagnosis. Please divided into two sentences when it is possible

According to the abovementioned criteria, 58 cases (78.4%) had at least one episode of remission (51 unreported). Overall mortality was 44 (46.3% of the reported cases; 30 unreported) patients.

**Statistical analysis**

Software used for data analyses was SPSS v.17.0 (SPSS corp., Chicago, Il, USA). Statistical differences between patients’ subgroups were performed by using χ2 and Fishers’ exact tests for proportions and the Students t test for continuous data. Survival analysis was done with life tables and Kaplan-Meier methods and log-rank test. All statistical tests were performed at the 0.05 significance level. 

## Results

Overall 125 recipients of heart allograft with lymphoproliferative disorder who had a verified history of either having or not having use of rituximab therapy were entered into the analysis. The list of the studies of their patients, which were finally included into analysis are summarized in table I. There were 78 (75.7%) males and 25 (24.3%) female (22 unreported) patients. Mean age at diagnosis of PTLD was 37.5±23.4 years. 30 (26.5%) of the patients were at the pediatric age (12 unreported). The mean interval between transplantation and the diagnosis of PTLD was 47.2 ± 39.0 months whereas follow up time after diagnosis of PTLD was 30.4±35.8 months. 

Table II summarizes comparative data of the study population regarding the history of rituximab therapy. As can be seen, patients who undergone rituximab therapy were significantly more likely to be younger at age, having more aggressive histopathological type of lymphomas, and also none of them receive induction therapy. At the last follow, 44 (46.3%) patients were dead (30 case unreported data). Survival analyses using either ‘death irrespective of the reason’ (P-value= 0.47) or ‘death due to PTLD’ (P-value= 0.27) as the outcome showed no significant difference regarding receiving rituximab therapy for heart recipients, neither any significant difference was found for remission rates between patients who had received rituximab and patients who had not (P-value= 0.637). In order to analyze a potential disparity between children and adults with regard to response to rituximab therapy, we reanalyzed the data for each age subgroup, separately. This time, survival analyses showed a significant beneficial effects for pediatric heart recipients with PTLD who get rituximab therapy (P-value= 0.031; figure 1), but no benefit was found for adult heart recipients with PTLD regarding rituximab therapy (P-value= 0.2). For patients with CD20 positive results, survival analysis, showed no survival benefit (P-value= 0.95); however, we should consider that CD20 positive patients who undergone rituximab therapy had a significantly more unfavorable tumoral histopathology (11(69%) monomorphic lesions versus 5 (31%) for those who did not get rituximab). 

**Table I:** List of the series included

**Studies **	**Frequency**	**Percent**
**Aigner et al. [18]**	1	.8
**Tsai et al.[19]**	2	1.6
**Dotti et al. [20]**	4	3.2
**Poirel et al. [21]**	14	11.2
**Trappe et al. [22]**	2	1.6
**Muti et al. [23]**	22	17.6
**Wasson [24]**	4	3.2
**Vakiani et al. [25]**	9	7.2
**Schubert et al. [26]**	6	4.8
**Oertel et al. [27]**	3	2.4
**Buadi et al. [28]**	4	3.2
**Douglas et al. [29]**	2	1.6
**Timms et al. [30]**	3	2.4
**Elad et al. [31]**	5	4.0
**Morovic [32]**	1	.8
**Peraira et al. [33]**	2	1.6
**Manlhiot et al. [34]**	2	1.6
**Pitman e al. [35]**	3	2.4
**Windebank et al. [36]**	3	2.4
**Burra et al. [37]**	10	8.0
**His et al. [38]**	2	1.6
**Benkerrou et al. [39]**	13	10.4
**Blaes [40]**	1	.8
**Oertel et al. [41]**	2	1.6
**Zimmermann et al. [42]**	1	.8
**Picarsic et al. [43]**	4	3.2
**Total**	125	100.0

**Table II:** Comparative data of the study cases and control population

**Variables **	**Rituximab **	**No rituximab**	**Sig.**	**Available data**
**Age (yr)**	30.3±24.3	40.4±22.4	0.034	113
**Pediatric; <18 yr/o (%)**	13 (39.4)	17 (21.3)	0.06	113
**Gender male (%)**	19 (73.1)	59 (76.6)	0.79	103
**Time to PTLD development (mo)**	44.5±39.8	48.2±38.9	0.66	118
Multi organ involvement (%)*	10 (41.7)	29 (39.3)	0.52	85
Disseminated PTLD (%) *	3 (17.6)	18 (30.5)	0.37	76
**EBV status (%)**	22 (81.5)	50 (75.8)	0.785	93
**Remission episode (%)**	20 (80)	38 (77.6)	0.53	74
**Monoclonal lesions vs. polyclonal (%)**	9 (90)	34 (68)	0.26	60
**Lymphoma cell type B cell (%)**	24 (96)	68 (90.7)	0.68	100
**Morphology **	1(3.1)	8 (11.6)	0.003	101
**Early lesion (Plasmacytic hyperplasia)**	3 (9.4)	23 (33.3)		
**Polymorphic B cell lymphoma**	27 (84.4)	31 (44.9)		
**Monomorphic PTLD**	1 (3.1)	7 (10.1)		
**Hodgkin lymphoma**	1(3.1)	8 (11.6)		

**Figure I:**
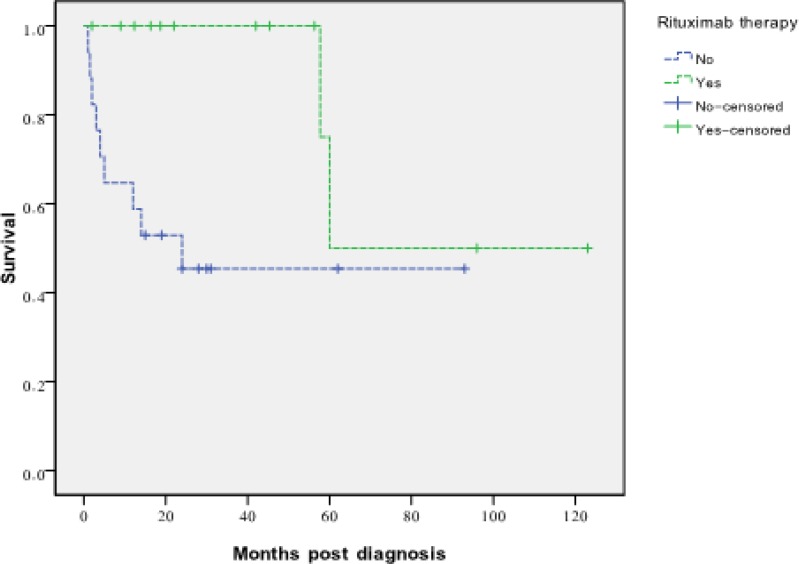
Survival curves of pediatric heart Recipients developing PLTD ,who have undergone rituximab therapy or have not

## Discussion

Rituximab is a chimeric anti-CD20 IgG monoclonal antibody which through its murine Fab domain binds to the CD20 antigen, a transmembrane protein located on the surface of mature B-cells. The CD20 antigen is one of regulators of transmembrane calcium conductance and cell-cycle progression during activation of B-cells. Rituximab was firstly approved for the treatment of relapsed CD20-positive non-Hodgkin lymphomas with reported high remission rates (44); and since then, it has been widely used alone or in combination with other agents for the treatment of various hematological malignancies (45-47).

Rituximab has also been used in the management of PTLD (48). Although due to its indiscriminate attack to CD20 cell, irrespective of their status as neoplastic or normal, several serious side effects are associated with this agent administration, in a way that some authors have doubted its safety as a drug. Data on the feasibility of rituximab administration in PTLD patients is quite more limited that it in non-transplant context. The current study which is based on cumulative data of 26 studies showed no significant beneficial effects for rituximab therapy in heart transplant recipients. Nonetheless, we should bring in mind that our analyses were not based on data achieved from a randomized controlled trial, and a selection bias in defining the patient population in our study is inevitable. For example, as it is summarized in table II, patients who had undergone rituximab therapy had a significantly worse histopathological feature of the tumor; this may in part show that why we did not observe a beneficial effect for the rituximab therapy; the drug has been used for patients of more aggressive tumors. 

The most important finding of the current study is that we found that rituximab therapy is associated with a significant superior survival for pediatric heart transplant recipients. Taking a precise look at the survival curves illustrated in the figure 1, no mortality has been occurred in heart transplant children who had received rituximab during the first 4-5 years after PTLD diagnosis. This finding is of utmost importance when we consider the fact that PTLD is highly fatal in heart graft recipients compared to recipients of other organs (49). This makes rituximab an indispensible drug to manage PTLD arising in heart transplant recipients. 

Findings of the current study have other clinical implications as well. Although both rituximab and chemotherapy have been approved to be used as the therapeutic options in PTLD patients, chemotherapy has been associated with high rates of toxicity related mortality. In one study over 1/4^th^ of all PTLD patients who had undergone chemotherapy died of its associated toxicity (50). Although this fact does not diminish the importance of chemotherapy in patients with more aggressive PTLD courses, use of rituximab therapy alone or in combination with low dose chemotherapy might provide higher survival rates, both due to lowering adverse effects of chemotherapy and therapeutic effects of rituximab. This idea would be more strengthen when we consider that compared to cytotoxic chemotherapy alone, a combination of chemotherapy and rituximab in the treatment of non-Hodgkin’s lymphoma is more effective (51). more specifically, a trial of six patients with PTLD has showed that a combination of chemotherapy with rituximab was associated with 100% remission rate in their series (52); and based on this finding authors have suggested that rituximab can also sensitize tumoral cells to the effects of chemotherapy. Moreover, there are studies demonstrating high response rate to rituximab therapy in the pediatric setting (53). Consistent to our findings, Webber et al. have proposed rituximab as the first line treatment for refractory PTLD developing in pediatric solid organ recipients (54). Putting all the above mentioned data together, we recommend that all pediatric heart transplant recipients who develop PTLD to receive rituximab therapy in their anti- tumoral therapy regimens, either alone or in combination with chemotherapy. For adult recipients of heart graft, more data are needed from randomized controlled trials to confirm its beneficial effects. 

This study has some limitations. These limitations are inevitable due to the nature of the PTLD. Int Surveys which includes its cases from different reports (55-71). Patients whose data were used for analysis in this study were gathered from different case reports or series, which might follow more or less inconsistent approaches. However, to deal with this problem we standardized data of different studies to be able to cumulate them all in a single database. On the other hand, our study represents the single largest patient population of heart transplant recipients with PTLD who undergone had rituximab therapy. Moreover, the very excellent outcome achieved by rituximab therapy in pediatric recipients of heart allograft with PTLD leaves rituximab a compelling agent in the management of this disease. 

In conclusion, this study showed that rituximab therapy in pediatric heart transplant recipients with PTLD represents surprisingly excellent results, making rituximab an indispensible agent in the management of the disease. However, in this study we found not a comparable result for adult recipients of cardiac transplant. The rationale behind this observation, we believe, lies on the methodology of the study and the selection bias between the patients who received the rituximab or not; because patients who received the agent were significantly more likely to have worse histopathological features for the tumor. To define feasibility of rituximab therapy in adult recipients of heart graft with PTLD, randomized controlled trials are needed. 
